# Correction: *Lonicera japonica* Thunb. as a promising antibacterial agent for *Bacillus cereus* ATCC14579 based on network pharmacology, metabolomics, and *in vitro* experiments

**DOI:** 10.1039/d4ra90032d

**Published:** 2024-04-09

**Authors:** Nan Xu, Li-hua Du, Yan-chao Chen, Jin-hao Zhang, Qian-feng Zhu, Rong Chen, Guo-ping Peng, Qi-ming Wang, Hua-zhong Yu, Li-qun Rao

**Affiliations:** a Hunan Engineering Laboratory for Good Agricultural Practice and Comprehensive Utilization of Famous-Region Medicinal Plants, Hunan Agricultural University Changsha China; b Key Laboratory of Hunan Forest Products and Chemical Industry Engineering, Jishou University Jishou China

## Abstract

Correction for ‘*Lonicera japonica* Thunb. as a promising antibacterial agent for *Bacillus cereus* ATCC14579 based on network pharmacology, metabolomics, and *in vitro* experiments’ by Nan Xu *et al.*, *RSC Adv.*, 2023, **13**, 15379–15390, https://doi.org/10.1039/D3RA00802A.

The authors regret errors in the original article in the details of the minimum inhibitory concentrations provided in the Results section and in the Discussion section.

The sentence beginning “Meanwhile, the minimum inhibitory concentrations (MIC) of luteolin…” in the Results section should read: “Meantime, the minimum inhibitory concentrations (MIC) of luteolin, quercetin, and kaempferol against *Bacillus cereus* ATCC14579 were 15.625 μg mL^−1^, 31.25 μg mL^−1^, and 15.625 μg mL^−1^, respectively.”

In addition, the sentence beginning “Meantime, the active ingredients luteolin…” in the Discussion section should read: “Meanwhile, the active ingredients luteolin (MIC = 15.625 μg mL^−1^), quercetin (MIC = 31.25 μg mL^−1^), and kaempferol (MIC = 15.625 μg mL^−1^) also inhibited *Bacillus cereus* ATCC14579 with good inhibitory effect.”

The conclusions presented remain unaffected by these changes.

The Royal Society of Chemistry apologises for these errors and any consequent inconvenience to authors and readers.

## Supplementary Material

